# Supplementary material for: Usefulness of Hospital Admission Chest X-ray Score for Predicting Mortality and ICU Admission in COVID-19 Patients
Source: J Clin Med. 2022 Jun 20;11(12):3548. doi: 10.3390/jcm11123548 (PMC9225367; doi:10.3390/jcm11123548)
Supplement: Supplementary file 1 [file jcm-11-03548-s001.zip › jcm-1718526-supplementary.pdf]

Supplementary Materials:

I. Description of analysis plan

A 4 steps analysis plan has been conducted as follows:

1. Evaluation of the agreement level among 3 different doctors in CXR scores estimation, by using Kendall's Coefficient of Concordance (W) and corresponding significance test (Kendall & Gibbons, 1990).

2. Exploratory data analysis: Conventional descriptive statistics and data visualization have been conducted to determine distribution characteristics of qualitative and quantitative parameters including CXR score among the subgroups of clinical outcomes (dead or survived, with or without ICU admission). Statistical inference on the difference in distribution of those parameters between groups were based on Mann-Whiney U test (for continuous data) and Pearson's Chi-squared test (for categorical data).

3) We performed a ROC analysis and a *post-hoc* optimization process to determine the optimal cut-off points of CXR score allowing for making prediction of dead and ICU admission event. the effect of having high CXR score on probabilities of occurrence of dead and ICU admission events was then estimated by non-parametric model Kaplan-Meier method and Log-rank test.

4) Time-to-event analysis has been conducted to investigate the contribution of CXR score as a predictor of mortality and ICU admission. A time-dependent ROC AUC analysis was applied to evaluate the performance of CXR-score to make prediction of survival outcome at a given time point, in comparison with other routine laboratory parameters.

Next, we applied the "Select K-best" algorithm to determine the most efficient combination of 1 to 5 predictors which allow for accurate prediction of time to the occurrence of event (dead and ICU admission) through a Cox-proportional hazard regression model, by optimizing the concordance index value of these models.

For main statistical inference on the marginal effect of CXR score on the risk of dead or ICU admission outcomes, we applied a Cox-PH regression model with CXR score and adjustment for patient's age.

All statistical analysis was performed using Python programming language with li-braries pandas [22], scipy [23], lifelines [24] (Survival analysis), matplotlib [25] (statis-tical graphics) and scikit-learn [26] (Select K best algorithm). Statistical inference was based on 95% confidence interval and null-hypothesis testing at significance threshold of $p < 0.05$.

II. Tables and Figures:

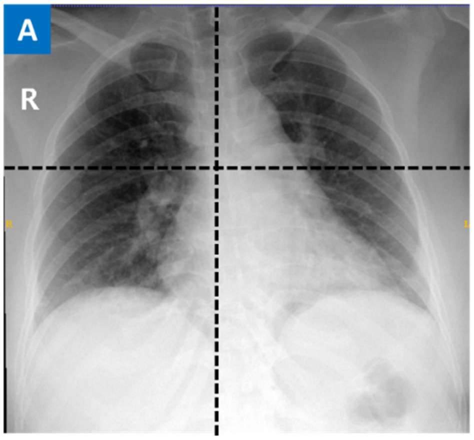

	Q1	Q2	Q3	Q4
Severity	2	1	1	1
Extent	1	1	1	1

Total = 9

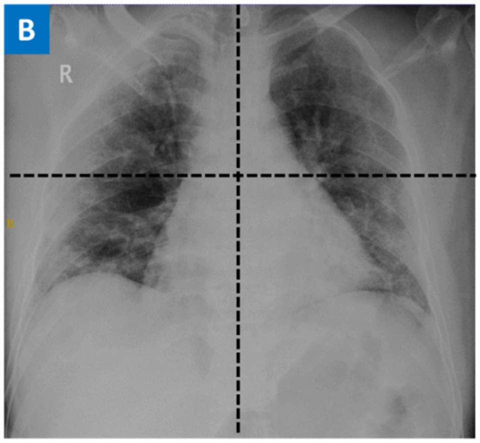

	Q1	Q2	Q3	Q4
Severity	3	3	2	2
Extent	2	2	2	2

Total = 18

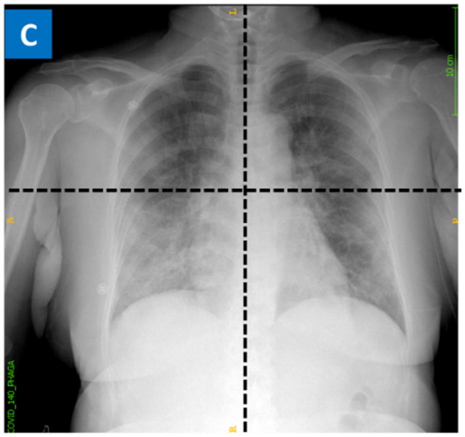

	Q1	Q2	Q3	Q4
Severity	1	2	1	1
Extent	3	3	3	3

Total = 17

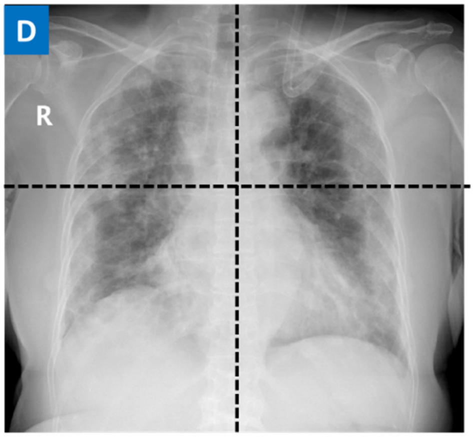

	Q1	Q2	Q3	Q4
Severity	2	2	2	2
Extent	3	3	2	3

Total = 19

Figure S1: Typical findings on chest X-ray and CXR scores in four clinical cases with pneumonia secondary to COVID-19.

A) 34-year-old female patient, BMI=30, admitted to the hospital on the 4th day after onset of the symptom, CXR1 score 9, breathing room air, discharged after 7 days, fully recovered.

B) 40-year-old male patient, BMI=28, admitted to the hospital on the 10th day after the onset, CXR1 score 18, severe disease progression with breathing on mask oxygen, discharged after 3 weeks, alive.

C) 69 years old female patient, BMI=24, hospitalized on the 10th day after onset, oxygen supply via bag mask, CXR1 score 17, discharged after 15 days, alive.

D) 71 years old female patient , BMI=27, hospitalized 10 days after onset, CXR1 score 19, full respiration support with tracheal intubation, continuous renal replacement therapy and died after 30 days of hospital stay.

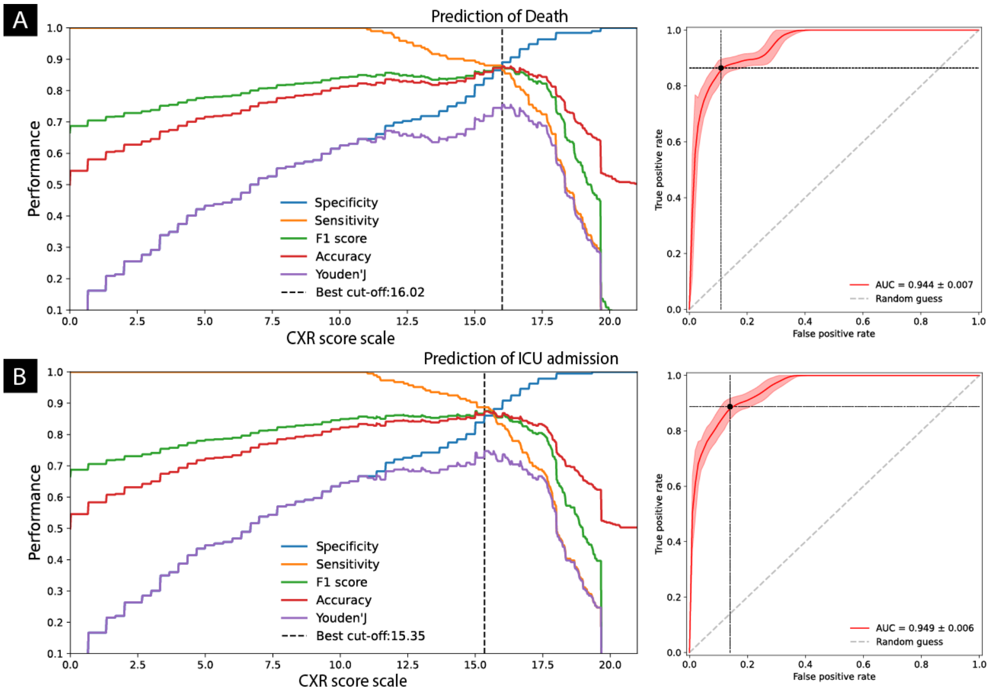

Figure S2: Prediction of Death (A) and ICU admission (B) based on CXR1 score.

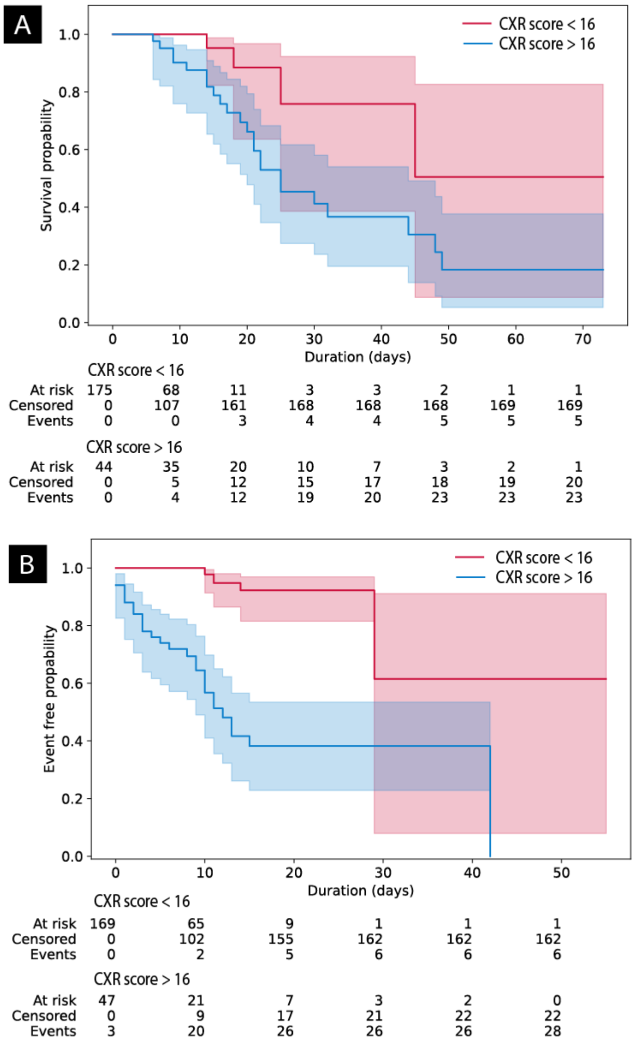

Figure S3: Survival function of Death (A) and ICU admission (B) outcome, stratified by CXR1 score best cutoff. Note that only in-hospital mortality is taken into account.

Table S1: Inte r-rater reliability of CXR scoring, measured by Kendall's W coefficient.

Parameters	Estimated Kendall's W	Bca95%CI*		P value
Total CXR score	0.904	0.886	0.919	<0.000001
Total Extent	0.89	0.874	0.905	<0.000001
Total Severity	0.855	0.830	0.874	<0.000001
Extent Score Q1	0.772	0.740	0.797	<0.000001
Extent Score Q2	0.794	0.770	0.816	<0.000001
Extent Score Q3	0.750	0.714	0.784	<0.000001
Extent Score Q4	0.792	0.770	0.811	<0.000001
Severity Score Q1	0.686	0.648	0.726	<0.000001
Severity Score Q2	0.717	0.682	0.749	<0.000001
Severity Score Q3	0.693	0.656	0.731	<0.000001
Severity Score Q4	0.737	0.710	0.763	<0.000001

*Note: Bca95%CI: 95% confidence interval, based on bootstrapping with bias correction accelerate estimation.

Table S2: Predictive performance of CXR-score at critical threshold of 16

Metric	Target : Death	ICU admission
Sensitivity	0.906	0.876
Specificity	0.864	0.881
F1 score	0.888	0.879
Balanced accuracy	0.885	0.879
PPV	0.870	0.881
NPV	0.902	0.877
LR+	6.692	7.409
LR-	0.108	0.140
Youden J index	0.771	0.758

Table S3A: Five best models with optimal concordance index values (Survival outcome)

Parameter	HR	Lower 95%CI	Upper 95%CI	P value
K=1 Model (Concordance index = 0.806)				
CXR score	1.274	1.086	1.495	0.003
K=2 (Concordance index = 0.826)				
CXR score	1.332	1.096	1.619	0.004
AST	1.004	1.001	1.006	0.008
K=3 (Concordance index = 0.839)				
CXR score	1.331	1.095	1.618	0.004
Diabetes	1.936	0.867	4.323	0.107
AST	1.004	1.001	1.007	0.002
K=4 (Concordance index = 0.846)				
CXR score	1.409	1.108	1.792	0.005
Age	1.045	1.007	1.083	0.018
Diabetes	1.641	0.725	3.715	0.235
AST	1.005	1.002	1.008	0.001
K=5 (Concordance index = 0.930)				
CXR score	1.817	1.104	2.992	0.019
Age	1.093	1.025	1.165	0.07
Diabetes	3.372	0.872	13.046	0.078
AST	1.007	1.001	1.013	0.014
CRP	1.009	0.994	1.023	0.235

Table S3B: Cox-PH model used for statistical inference

Parameter	HR	Lower 95%CI	Upper 95%CI	P value
CXR score	1.333	1.097	1.621	0.004
Age	1.046	1.012	1.081	0.008

Note: Concordance index = 0.825; Partial AIC = 179.988 ; log-likelihood ratio test = 24.119 on 2 dof

Table S4A: Five best models with optimal concordance index values (ICU admission event)

Parameter	HR	Lower 95%CI	Upper 95%CI	P value
K=1 Model (Concordance index = 0.924)				
CXR score	1.645	1.374	1.970	<0.0005
K=2 (Concordance index = 0.927)				

CXR score	1.690	1.387	2.059	<0.0005
Lymphocyte count	0.821	0.278	2.421	0.721
K=3 (Concordance index = 0.927)				
CXR score	1.784	1.439	2.211	<0.0005
Lymphocyte count	0.910	0.324	2.551	0.857
AST	1.001	0.998	1.004	0.437
K=4 (Concordance index = 0.939)				
CXR score	2.099	1.477	2.981	<0.0005
Lymphocyte count	1.094	0.253	4.732	0.904
AST	1.002	0.999	1.006	0.244
CRP	1.001	0.988	1.014	0.872
K=5 (Concordance index = 0.939)				
CXR score	2.214	1.496	3.277	<0.0005
Lymphocyte count	1.145	0.273	4.800	0.853
AST	1.003	0.999	1.007	0.158
ALT	0.998	0.993	1.003	0.485
CRP	1.001	0.998	1.014	0.906

Table S4B: Cox-PH model used for statistical inference

Parameter	HR	Lower 95%CI	Upper 95%CI	P value
CXR score	1.641	1.369	1.968	<0.0005
Age	1.003	0.978	1.029	0.807

Note: Concordance index = 0.925; Partial AIC = 235.008 ; log-likelihood ratio test = 77.674 on 2 dof

Table S5: Time-dependent ROC AUC values

Predictors	Mean	SD	Median	95%CI	
**CXR score**	0.856650	0.101879	0.852926	0.694053	1.000000
**Age**	0.784137	0.101710	0.801440	0.637957	0.932353
**AST**	0.730847	0.107362	0.739748	0.593766	0.891524
**CRP**	0.647378	0.194162	0.615858	0.405741	1.000000
**Neutrophyle count**	0.611244	0.256740	0.602553	0.289952	1.000000
**White blood cell count**	0.586572	0.254557	0.565309	0.276848	0.998227
**BMI**	0.579946	0.095998	0.571754	0.457183	0.748322
**ALT**	0.520494	0.086537	0.492695	0.394359	0.646561
**Heart rate**	0.520084	0.200351	0.574558	0.174955	0.782166
**Platelet count**	0.511349	0.275268	0.433485	0.215228	0.978723
**Temperature**	0.503226	0.138383	0.468085	0.380849	0.795342
**Lymphocyte count**	0.416151	0.247108	0.451900	0.086843	0.842831
**SpO2**	0.384023	0.180667	0.336175	0.111625	0.620829
